# Lifetime Impact Study for Achondroplasia (LISA): Findings from an observational and multinational study focused on health-related quality of life in individuals with achondroplasia in Latin America

**DOI:** 10.1016/j.gimo.2023.100843

**Published:** 2023-11-18

**Authors:** Juan Llerena, Pablo Rosselli, Amanda Aragão, Cristina Valenzuela, Debora Bertola, Yaneth Mendez, Mariana del Pino, Nicolette Calvacanti, Paula Thomazinho, Jeanne M. Pimenta, Shelda Cohen, Tom Butt, José C. Thomaz, Renée Shediac, Richard Rowell, Tatiana S.P.C. Magalhães, Chong Kim, Virginia Fano

**Affiliations:** 1 Instituto Fernandes Figueira, Fundação Oswaldo Cruz, Rio de Janeiro, Brazil; 2 Fundación Cardioinfantil, Instituto de Cardiología, Bogotá, Colombia; 3 Instituto da Criança, Hospital das Clínicas da Faculdade de Medicina da Universidade de São Paulo, São Paulo, Brasil; 4 Hospital de Pediatría “Prof. Dr. Juan P. Garrahan,” Buenos Aires, Argentina; 5 BioMarin (UK) Limited, London, United Kingdom; 6 BioMarin Pharmaceutical Inc, Novato, CA; 7 BioMarin Farmacêutica do Brasil Ltda, São Paulo, Brazil

**Keywords:** Achondroplasia, Disease burden, Health-related quality of life, Health care resource use, Skeletal dysplasia

## Abstract

**Purpose:**

The multisystem clinical manifestations and complications of achondroplasia, the most common form of disproportionate short stature, can cause functional impairment and psychosocial burden. The Lifetime Impact Study for Achondroplasia (LISA), aimed to assess health-related quality of life and medical resource utilization among Latin America patients with achondroplasia.

**Methods:**

Data were collected from individuals aged 3 years and above in Argentina, Brazil, and Colombia between 2018 and 2021. A total of 172 patients participated in the study.

**Results:**

Children with achondroplasia reported lower scores compared with average stature children in Quality of Life in Short Stature Youth (QoLISSY) and Pediatric Quality of Life Inventory (PedsQL) questionnaires, with the greatest impact on physical and social domains. Among adolescents, a significant percentage reported pain, 10.3% experienced pain in 3 or more sites. Adults scored lower than the reference population in the EQ-5D-5L Visual Analog Score, and a considerable portion reported moderate to severe anxiety/depression, pain or discomfort, and mobility problems. The Nottingham Health Profile (NHP) revealed poor health status in terms of energy, pain, and mobility. Medical events, particularly musculoskeletal and connective tissue disorders were reported, resulting in high medical resource utilization.

**Conclusion:**

Overall, the Lifetime Impact Study for Achondroplasia study provides extensive data on health-related quality of life, psychosocial impact, and health care resource utilization among individuals with achondroplasia in Latin America. The findings confirm a significant burden of illness across multiple domains for these individuals.

## Introduction

Achondroplasia is an autosomal dominant genetic disorder caused by pathogenic variants in fibroblast growth factor receptor 3 (*FGFR3;* HGNC: 3690) gene.^1^^,^^2^ This skeletal dysplasia is the most common form of disproportionate short stature.^1^ Worldwide, the disease occurs approximately in 1 in 25,000 live births^3^ and the prevalence in Latin America (LATAM) is 2.5 to 4.6 per 100,000 individuals.^3^^,^^4^

Achondroplasia is associated with impaired development of the bones and cartilage.^3^ Patients typically present with disproportionate short stature, rhizomelic limb shortening, tibial bowing, narrow chest, macrocephaly with prominent forehead, midface hypoplasia, and a depressed nasal bridge.^5^^,^^6^ Achondroplasia-related complications and comorbidities vary at different life stages.^7^, ^8^, ^9^ More prevalent complications related to skeletal manifestations are genu varum and thoracolumbar kyphosis, occurring in infancy and childhood.^7^, ^8^, ^9^ In infants, there is also a high prevalence of neurological and ear, nose, and throat (ENT) symptoms including spinal stenosis or compression, foramen magnum stenosis, hypotonia, and sleep-disordered breathing.^7^, ^8^, ^9^ The neurological manifestations in the arms, neck, and legs continue in adolescence.^7^, ^8^, ^9^ Pain is reported in all age groups, and cardiovascular conditions and obesity are more common in adulthood.^7^, ^8^, ^9^

Because of the multisystem and lifelong effects of achondroplasia-related symptoms and comorbidities, international and LATAM guidelines recommend a multidisciplinary approach for patient management, which includes medical, functional and psychosocial care throughout life.^10^^,^^11^ As consequences of the clinical manifestations of achondroplasia, the developmental milestones of children living with the disease differ from average stature individuals.^8^ The short stature, shortened limbs, ligamentous laxity, and communication issues caused by hearing and speech limitations impair or delay early developmental milestones and independence from parents or caregivers, which, in turn, can result in reduced functionality, limitations in the ability to perform daily activities, poor self-esteem, and reduced quality of life (QoL).^12^, ^13^, ^14^ Previous studies have reported limited functionality as the main generators of child/adolescent and parental anxiety and stress according to patients and their caregivers in the United States and Spain.^15^^,^^16^

Although there is a consensus that achondroplasia greatly affects patients’ physical functioning and psychosocial life, data related to psychosocial status of the adult achondroplasia population provide inconsistent findings. A recently published study with European patients of a broad range of ages, including adults, demonstrated that achondroplasia associates with impaired QoL and functionality, particularly related to physical domains.^17^ Some reports even show, by using patient-reported health-related QoL (HRQoL) surveys, that patients’ mental scores are similar to standard values found in general population, meanwhile the physical component score is significantly reduced.^18^, ^19^, ^20^ However, in these studies, the higher-than-expected scores in the physical and mental components are not necessarily representative of the general achondroplasia population. The higher than expected mental component scores can be explained by the fact that the majority of the participants in both of the studies belonged to, and were supported by, patient’s associations that contributed to healthier mental function, whereas the decline in physical scores is usually associated with musculoskeletal complaints in elderly population, a cohort found in a reduced number in these studies.^18^^,^^19^ Data from other studies, such as that by Jennings et al and Gollust et al, describe reduced QoL and significant levels of depression and anxiety in adults with achondroplasia, which was correlated with chronic pain.^12^^,^^20^

Although there are a number of studies on the natural history of achondroplasia and its psychosocial burden, to date, there is a lack of published data on the lifetime burden associated with living with the condition, especially in the LATAM population. Of note, a recently published systematic literature review evidenced the high burden of achondroplasia on affected individuals and on health care resources in LATAM countries.^4^ However, the interpretation and comparison of outcomes was confounded/limited by the heterogeneity of the clinical and methodological characteristics of the studies, mainly because of small sample sizes.

The Lifetime Impact Study for Achondroplasia **(**LISA; NCT03872531) was an observational study that aimed to describe and quantify the lifetime impact of achondroplasia in affected individuals in LATAM. Here, we report the findings from LISA, describing the clinical and psychosocial burden, HRQoL, and health care resource use of a LATAM population of patients with achondroplasia.

## Materials and Methods

### Study design

The LISA (NCT03872531) was a multinational, observational, retrospective study with a cross-sectional component assessing HRQoL among individuals living with achondroplasia in LATAM.

The study took place in 4 sites across 3 LATAM countries, Argentina, Brazil, and Colombia. All the sites provide multidisciplinary consultancy for patients with achondroplasia.

### Sample population

Table 1 shows the main characteristics of the LISA study population. The study enrollment occurred from 2 January 2018 to 16 July 2021. Enrollment into the study was divided by age groups as detailed in Table 1. The number of participants included who had limb lengthening surgery was limited to 20% to minimize selection bias.

**Table 1:** LISA participants by age, country, and/or analysis subgroups

LISA Participants	*n* (%)
Number of recruited patients per age group (years)	175 (100)
3-5	20 (11.4)
6-10	30 (17.1)
11-15	30 (17.1)
16-20	20 (11.4)
21-30	20 (11.4)
31-40	20 (11.4)
≥41	35 (20.0)
Number of enrolled patients	172 (100)
Argentina	37 (21.5)
Brazil	94 (43.0)
Colombia	41 (23.8)
Discontinuations	0 (0)
Study analysis sets	
Enrolled Analysis Set (EAS)^a^	172 (100)
Pediatric History Analysis Set (PHAS)^b^	32 (18.2)
Historical data (PHAS)^c^ by age (years)	32 (100)
3	4 (12.5)
4	5 (15.6)
5	5 (15.6)
6	2 (6.3)
7	4 (12.5)
8	0 (0.0)
9	2 (6.3)
10	0 (0)
>10	10 (31.3)

*EAS*, Enrolled Analysis Set; *PHAS*, Pediatric History Analysis Set.

a EAS, Enrolled Analysis Set—all enrolled patients.

b PHAS, Pediatric History Analysis Set—patients with at least a 3-year complete medical history before 18 years of age.

c The earliest date of medical history entered into the study database before enrollment.

The inclusion criteria for the study were as follows: ≥3 years of age at time of enrollment, have a documented diagnosis of achondroplasia based on genetic or clinical/radiological findings, cognitive and linguistic capacities necessary to complete questionnaires in the language of his/her country (and/or parents/legal representatives, as applicable), and have medical records available for at least the 3 years before the date of enrollment. The exclusion criteria were as follows: current or prior participation (in the previous 6 months) in any clinical trial of a medicinal product or medical device or in any BioMarin study. It should be noted that not all patients had genetic confirmation because this was not a requisite inclusion criterion.

### Data collection, management, and statistical analysis

Data about QoL, psychosocial and socioeconomic factors, and health care resource use were collected using questionnaires validated for local use. An electronic data capture (EDC) system (Classic Rave EDC 2020.2.0) was developed to receive the data collected in this study, including data entry screens and automated edit and logic checks to validate test data. The data collected from medical records and questionnaires were entered into the EDC system by trained study staff or a designee.

For data analysis, the population was divided into 2 sets, as displayed in Table 1: the Enrolled Analysis Set (EAS, *n* = 172), which included all enrolled participants who had consented to participate and met the inclusion criteria and was used for the study disposition, protocol deviations, demographic, and medical history summaries and analysis; and the Pediatric History Analysis Set (PHAS, *n* = 32), which included all participants with at least a 3-year complete medical history (meaning that all, or the vast majority, of medical records were available for the period) before 18 years of age. The PHAS population was used to analyze events related to medical history, health care resource use, and concomitant medication. The PHAS population were all from Argentina because this site had access to a comprehensive set of medical records.

The analyzed data were stratified by patient age (3-5, 6-10, 11-15, 16-20, 21-30, 31–40, and ≥41 years). Statistical analysis was carried out using SAS Institute Inc 2013 SAS/ACCESS 9.4 Interface to Adaptable Database System. The continuous variables were described by their mean, standard deviation, median, quartiles 1 and 3, extreme values (minimum and maximum), and the number of missing data items. Categorical variables were described (counts) by the absolute and relative (%) frequency of each class and the number of missing data items.

### Patient- and parent-reported outcomes questionnaires

At the time at enrollment, health-related questionnaires were completed related to the self-perceived impact of achondroplasia in daily life by the participants, or by their parents/caregivers/legal representatives in the case of minors. The questionnaires were divided into child/adolescent—Pediatric Quality of Life Inventory Questionnaire (PedsQL), Quality of Life in Short Stature Youth (QoLISSY), Pediatric Functional Independence Measure (WeeFIM), Adolescent Pediatric Pain Tool (APPT), and adult—5-level EQ-5D version of EuroQoL (EQ-5D-5L), Nottingham Health Profile (NHP), Brief Pain Inventory - Short Form (BPI-SF), and Work Productivity and Activity Impairment (WPAI). The outcome domains assessed in each one is shown in Supplemental Table 1. The questionnaires were given to the participants during a routine hospital visit or sent to his/her home, with the exception of the WeeFIM, which was applied by an investigator during a hospital visit or at a mutually convenient time over the telephone.

The scores obtained were compared with values found for the reference population, average stature individuals, or participants with other conditions affecting height, when available.

## Results

### Demographic and baseline characteristics of the patients with achondroplasia

The study enrolled a total of 172 participants: 37 in Argentina (1 site), 94 in Brazil (2 sites), and 41 in Colombia (1 site) (Tables 1 and 2). All enrolled participants met the inclusion criteria and completed the study. The demographic and baseline characteristics at the time of enrollment are described in Table 2. The age subgroups and gender distribution were homogenous, as predicted by the study design, with 81 (47.1%) males and 91 (52.9%) females with a median age across both genders of 16.0 years (Table 2). Similar to the EAS, the Argentinean PHAS population had a relatively equal gender distribution, 18 (56.3%) males and 14 (43.8%) females (Table 2). However, as only pediatric data were included, as expected, the median age was younger (12.0 years old). For the 8 (25%) participants aged ≥18 years at baseline from the PHAS, only data from their pediatric medical history were used in the analysis.

**Table 2:** Demographic characteristics at time of enrollment of patients with achondroplasia

Demographic Characteristics	Enrolled Analysis Set (EAS)^a^				Pediatric History Analysis Set (PHAS)^b^
	Brazil	Argentina	Colombia	Overall	Argentina
Participants per country, *n* (%)	94 (54.6)	37 (21.5)	41 (23.8)	172 (100.0)	32 (100.0)
Gender, *n* (%)					
Male	45 (47.9)	19 (51.4)	17 (41.5)	81 (47.1)	18 (56.3)
Female	49 (52.1)	18 (48.6)	24 (58.5)	91 (52.9)	14 (43.8)
Age (years)					
Median (25th, 75th Percentile)	18.5 (9.0, 32.0)	15.0 (7.0, 23.0)	16.0 (10.0, 32.0)	16.0 (8.0, 31.5)	12 (6.5, 17.5)
Min, Max	3, 65	3, 50	3, 71	3, 71	3, 43
Age subgroups (years)^c^, *n* (%)					
3-5	11 (11.7)	5 (13.5)	4 (9.8)	20 (11.6)	5 (15.6)
6-10	18 (19.1)	9 (24.3)	8 (19.5)	35 (20.3)	9 (28.1)
11-15	16 (17.0)	6 (16.2)	7 (17.1)	29 (16.9)	6 (18.8)
16-20	5 (5.3)	6 (16.2)	5 (12.2)	16 (9.3)	6 (18.8)
21-30	15 (16.0)	5 (13.5)	5 (12.2)	25 (14.5)	4 (12.5)
31-40	17 (18.1)	1 (2.7)	5 (12.2)	23 (13.4)	1 (3.1)
41 or more	12 (12.8)	5 (13.5)	7 (17.1)	24 (14.0)	1 (3.1)
Limb lengthening before enrollment^d^, *n* (%)					
Yes	2 (2.1)	3 (8.1)	7 (17.1)	12 (7.0)	3 (9.4)
Time since limb lengthening (years)^e^					
Median (25th, 75th Percentile)	5.2 (1.9, 8.5)	11.6 (2.8, 29.2)	6.0 (2.3, 8.7)	6.4 (2.6, 10.2)	11.6 (2.8, 29.2)
Min, Max	2, 9	3, 29	1, 18	1, 29	3, 29

*EAS*, Enrolled Analysis Set; *PHAS*, Pediatric History Analysis Set.

a All enrolled patients.

b Patients with at least a 3-year complete medical history before 18 years of age.

c When date of birth is missing or only the year is provided, age collected by the electronic case report form (eCRF) is used.

d Assessment closest to enrollment date.

e Time (years) since limb lengthening surgery (date of enrollment, earliest date of limb lengthening surgery)/365.25.

In total, 12 (7%) individuals from EAS underwent limb lengthening before enrollment, with a median time since surgery of 6.4 years (Table 2). Notably, in Colombia, a greater proportion of individuals had undergone a limb lengthening procedure (*n* = 7 of 41, 17.1%), compared with 3 of 37 participants (8.1%) in Argentina and 2 of 94 participants (2.1%) in Brazil. In the PHAS population, only a single male pediatric and 2 female adult participants had undergone limb lengthening.

In the EAS population, achondroplasia diagnosis was generally made very early in life, with a median age at diagnosis of 0.7 months. Some participants were diagnosed much later in life, with 1 participant in Brazil being diagnosed at 46.9 years. This variability in time to diagnosis is reflected by the mean (SD) age at diagnosis of 26.4 (80.85) months. Diagnostic practice differed between countries, with patients in Argentina being much more likely to have their diagnosis confirmed by genetic testing (75.7% of participants, compared with 40.4% in Brazil and 17.1% in Colombia). Genetic testing was not used as the initial method of diagnosis in Argentina and only very occasionally in Brazil and Colombia. Among the individuals with a genetic test, the most common *FGFR3* gene pathogenic heterozygous variants were c.1138G>A (*n* = 61, 91.0%) and c.1138G>C (*n* = 3, 4.5%), which was consistent across the 3 countries.

### Patient- and parent-reported outcomes

#### Children and adolescent QoL and psychosocial burden

**Figure 1:**
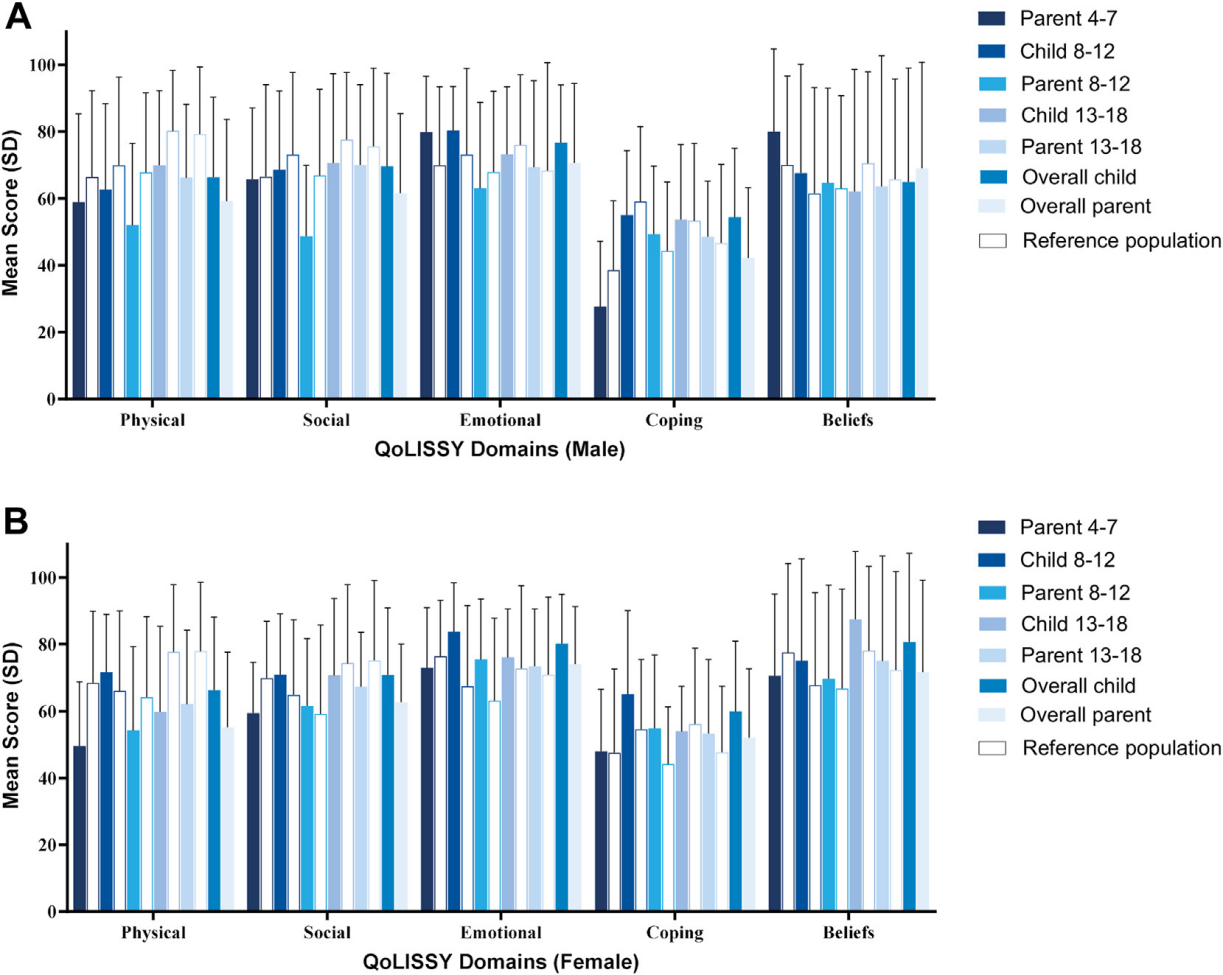
**Quality of Life in Short Stature Youth (QoLISSY) scores for male and female child/adolescent achondroplasia participants.** Child- and parent-perceived mean scores related to physical, social, emotional, coping, and beliefs domains for male (A) and female (B) patients with achondroplasia in comparison to the reference population (empty bars; average stature children). The QoLISSY scores ranged from 0 (poor quality of life) to 100 (better quality of life) and are presented as the mean score and its standard deviation (SD). QoLISSY, Quality of Life in Short Stature Youth.

The QoLISSY questionnaire evaluated child- and/or parent-perceived scores related to physical, social, emotional, coping, and beliefs domains, with a possible score of 0 (poor QoL) to 100 (better QoL). A total of 87 (96.6%) of 90 participants completed the QoLISSY questionnaire. The total QoLISSY score for questionnaires completed by patients (mean [SD] = 71.5 [17.5]) were higher than that for parent proxies (mean [SD] = 63.8 [18.9]), and the scores for patients and parents were lower than that for the reference population of average stature children (83.8 [12.7] and 82.7 [12.7], respectively). For the individual domains, patients with achondroplasia scored lowest in physical and social domains (Figure 1). In general, there were only small variations in the scores across age groups (Figure 1). In comparison with the average stature children, the scores of patients with achondroplasia are comparable or slightly lower in most domains (the largest score difference was 10 points), regardless of the gender or age range (Figure 1).

**Figure 2:**
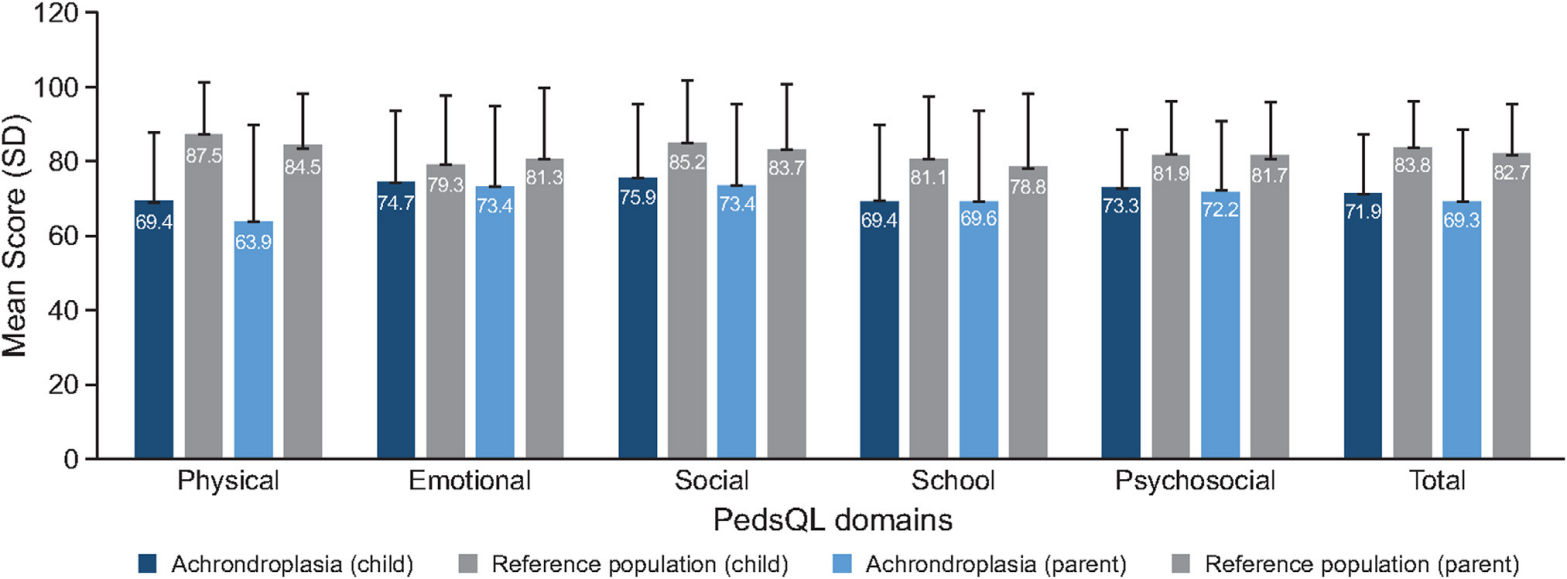
**Pediatric Quality of Life Inventory (PedsQL) for the child/adolescent achondroplasia participants.** Child- and parent-perceived scores (represented in dark and light blue bars, respectively) related to physical, emotional, social, school, and psychosocial functioning domains, as well as the total score compared with the reference population (gray bars, average stature children or proxy reference population [parent]). Scores on the PedsQL domains range from 0 (poor HRQoL) to 100 (better HRQoL). Data are presented as the mean score and its standard deviation (SD). PedsQL, Pediatric Quality of Life Inventory.

A second questionnaire was used to evaluate HRQoL and psychosocial burden in the achondroplasia child/adolescent population, the PedsQL, which rates child- and parent-perceived physical, emotional, social, school, and psychosocial functioning. Individual domains and total score values for the child version of the questionnaire were similar across Argentina, Brazil, and Colombia (Supplemental Figure 1). The total PedsQL score was slightly lower according to parents’ reports compared with the child version, with mean values of 71.9 for children and 69.3 for parents (Figure 2). Parents- and child-perceived scores for the individual domains were similar and all domains scored lower than the reference population (average stature children), particularly for the physical domain (Figure 2). The highest scores in the PedsQL for the patients with achondroplasia were in the emotional and social domains (Figure 2).

The functional skills and performance in daily activities in the children from 5 to 17 years old were measured using the WeeFIM instrument.^21^^,^^22^ WeeFIM is a questionnaire with 18 items that cover self-care, mobility, and cognition domains and assesses the need for assistance and the severity of disability, with higher scores indicating greater independence.^21^^,^^22^ The individual scores of the 3 evaluated domains, as well as the overall mean total scores, were similar across Argentina, Brazil, and Colombia (Supplemental Figure 2). The overall WeeFIM score increased with age, suggesting that older children (aged 13-17 years old) are more independent in terms of self-care, mobility, and cognition (Table 3). Despite the small number of patients that had the limb lengthening surgery, scores were higher for this group when compared with the overall achondroplasia children (Table 3), especially for 2 participants who had undergone upper limb (humerus) surgery and reported the highest self-care scores.

**Table 3:** Pediatric Functional Independence Measure (WeeFIM) scores for overall achondroplasia child/adolescent population and patients who underwent limb lengthening surgery by age group

WeeFIM Domain Scores	Children/Adolescent; Mean (SD)				Children/Adolescent With Limb Lengthening Surgery; Mean (SD)			
	Age: 5-17 (*n* = 89)	Age: 5-7 (*n* = 22)	Age: 8-12 (*n* = 31)	Age: 13-17 (*n* = 27)	Age: 5-17 (*n* = 7)	Age: 5-7 (*n* = 0)	Age: 8-12 (*n* = 3)	Age: 13-17 (*n* = 4)
Self-care^a^	32.1 (5.73)	37.4 (8.36)	49.8 (6.84)	54.6 (2.94)	53.8 (5.31)	-	51.7 (7.51)	56.0 (0.00)
Mobility^b^	31.6 (6.52)	28.4 (5.64)	33.6 (2.55)	34.7 (0.74)	35.0 (0.00)	-	35.0 (0.00)	35.0 (0.00)
Cognition^b^	32.1 (5.73)	30.1 (6.78)	33.8 (2.94)	34.8 (0.61)	33.7 (3.27)	-	32.3 (4.62)	35.0 (0.00)
Motor^b^	76.8 (16.79)	65.8 (10.36)	83.5 (8.51)	89.3 (3.46)	88.8 (5.31)	-	86.7 (7.51)	91.0 (0.00)
Overall score^c^	109.9 (20.74)	95.9 (15.34)	117.2 (9.19)	124.1 (3.51)	122.5 (8.57)	-	119.0 (12.12)	126.0 (0.00)

*WeeFIM*, Pediatric Functional Independence Measure.

a Range from 8 to 56.

b Range from 5 to 40.

c Range from 18 to 126. Lower scores indicate “Total assistance” and higher scores “Complete independence.”

#### Adult QoL, psychosocial, and socioeconomic burden

The HRQoL and psychosocial and socioeconomic burden because of achondroplasia was assessed in adults from the EAS population.

**Figure 3:**
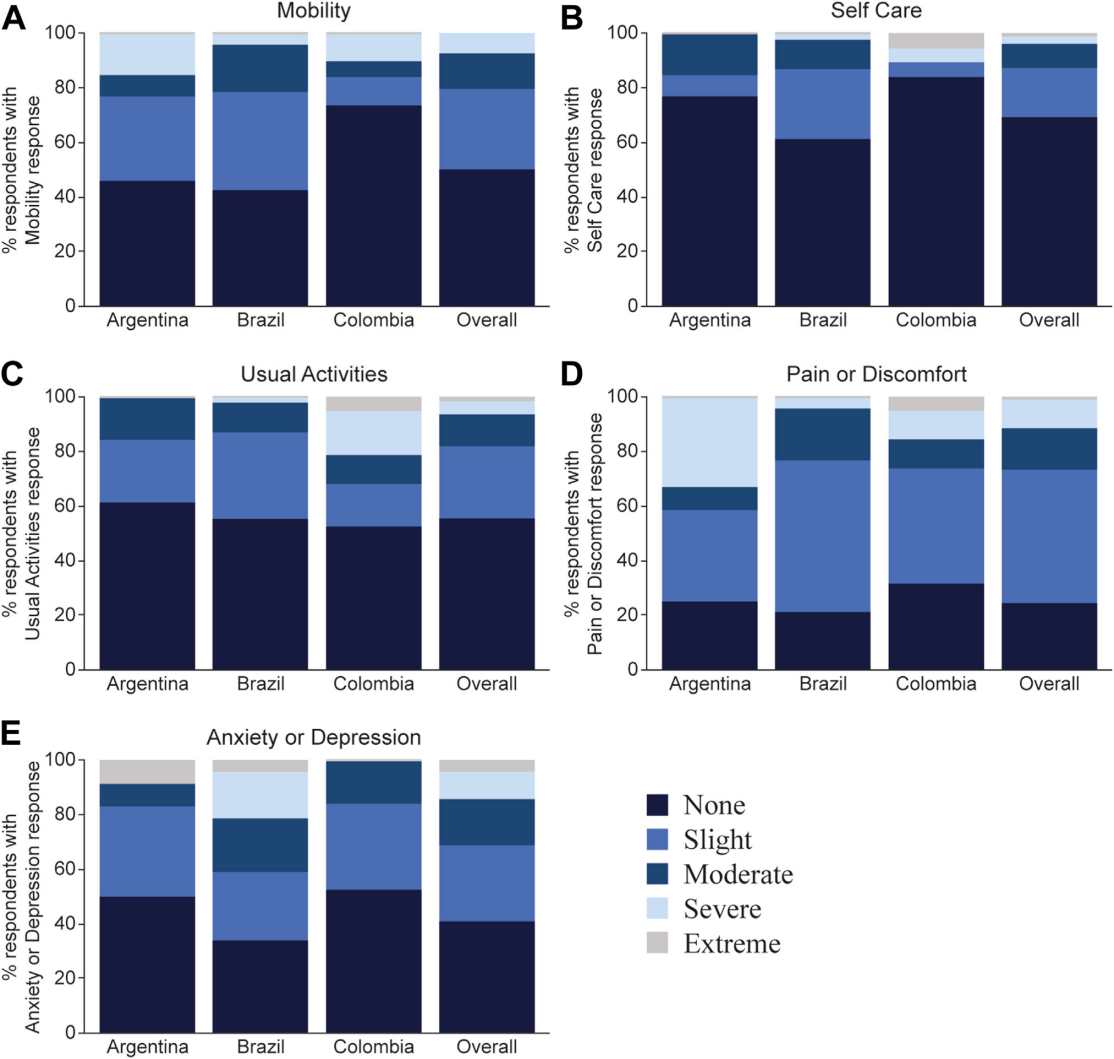
**Individual dimensions of the EuroQoL (EQ-5D-5L) in adult achondroplasia participants.** Percentage of adults reporting different levels (from none to extreme, according to the colored legend) of mobility (A), self-care (B), usual activities performance issues (C), feeling of pain or discomfort (D), and anxiety or depression (E) by country and the overall population.

These parameters were initially evaluated through the EuroQoL (EQ-5D-5L) questionnaire, an instrument that measures health status based on mobility, self-care, usual activities, pain/discomfort, and anxiety/depression dimensions, through a visual analog scale (VAS, measuring overall self-rated health status and ranging from 0, the worst health, to 100, the best health) and the Utility Index Score (UIS, assesses overall health status and ranges from −0.6, poorest health, to 1.0, better health). All adults completed the EQ-5D-5L. Of the 79 adult participants, 16 (20.3%) had moderate to severe problems with mobility (walking), 10 (12.7%) with self-care (washing or dressing themselves), and 14 (17.8%) with doing their usual activities (Figure 3A-C, respectively). Moreover, 21 (26.6%) reported moderate or severe anxiety/depression, and 20 (25.3%) reported moderate to severe pain or discomfort (Figure 3D and E, respectively). Despite these results, the mean (SD) EQ-5D-5L VAS score was lower than the unaffected population, 72.7 (20.1) versus 80.1 (25.3); and the UIS values of the achondroplasia and reference populations were comparable, 0.9 (1.4) versus 0.9 (0.2) (Supplemental Figure 3). The mean VAS score was lowest in Brazil (mean, SD of 68.3, 18.1) and highest in Colombia (mean, SD of 83.6, 20.5) (Supplemental Figure 3), reflecting the higher rates of anxiety and depression reported in Brazil (Figure 3C), whereas the UIS values were similar across countries (Supplemental Figure 3). Although 5 adult participants had undergone limb lengthening surgery, the overall mean VAS score (72.7) was similar to the non-limb-lengthened population.

The NHP was used to assess energy, pain, emotional reactions, sleep, social isolation, and physical mobility. Individual and total scores range from 0 (good subjective health status) to 100 (poor subjective health status). Seventy-seven (97.5%) adults completed the NHP. The mean total score was 25.8 (SD 21.59) and was similar among the countries, ranging from a mean of 25.1 (19.81) in Brazil to 27.0 (24.36) in Colombia. The highest scores, that indicate poor health status, were for energy, pain, and mobility, with respondents stating that they found it hard to stand for long (*n* = 47, 59.5%), hard to reach for things (*n* = 46, 58.2%), lost energy quickly (*n* = 45, 57.0%), were unable to wake up early (*n* = 38, 48.1%), had pain when walking (*n* = 37, 46.8%), had trouble getting up and down the stairs (*n* = 36, 45.6%), and found it hard to get dressed by themselves (*n* = 17, 21.5%). Five adults who had undergone limb lengthening surgery scored higher than the general adult population, with a mean (SD) of 40.6 (24.17), indicating poor subjective health, especially in the energy and social isolation domains. The individuals scores for general adult and limb-lengthened population are detailed in Table 4.

**Table 4:** Nottingham Health Profile (NHP) of achondroplasia adult population and patients who underwent limb lengthening surgery

Achondroplasia Patients	Mean (SD) Scores for NHP Individual Domains^a^					
	Energy	Pain	Emotion	Sleep	Social Isolation	Physical Mobility
Adults (*n* = 79)	35.4 (36.54)	29.3 (29.61)	22.5 (22.95)	25.6 (29.98)	12.8 (21.50)	29.3 (26.30)
Limb lengthening surgery (*n* = 5)	64.8 (48.94)	39.3 (39.44)	33.8 (6.16)	30.3 (32.15)	34.7 (11.59)	41.0 (34.93)

a Individual and total scores range from 0 (good subjective health status) to 100 (poor subjective health status).

The WPAI questionnaire was used to investigate the extent to which achondroplasia affects employment, work productivity, the ability to work, and the ability to perform regular daily activities other than work. All the 79 adult participants completed the questionnaire and 47 (59.5%) reported being employed. The percentages of employment varied widely between countries, with Argentina reporting only 1 (7.7%) patient as employed against 36 (76.6%) Brazilian adults employed. The mean number of hours worked was 27.4 (SD 16.94), and the perceived impact of achondroplasia on work productivity was 1.3 (2.40) on a scale from 0 (no effect) to 10 (high effect). Although the patients reported having little time off work because of achondroplasia (1.4% of work time missed), a greater proportion of participants reported being impaired at work (13.8%) or during regular daily activities (23.1%).

#### Pain in children/adolescent, adult, and overall achondroplasia population

In order to investigate the impact of pain and discomfort in patients with achondroplasia, 2 pain-related questionnaires, the APPT (for adolescent participants aged 8 to 17 years) and the Brief Pain Inventory (BPI, for adult participants with 18 years old or more) were utilized.

**Figure 4:**
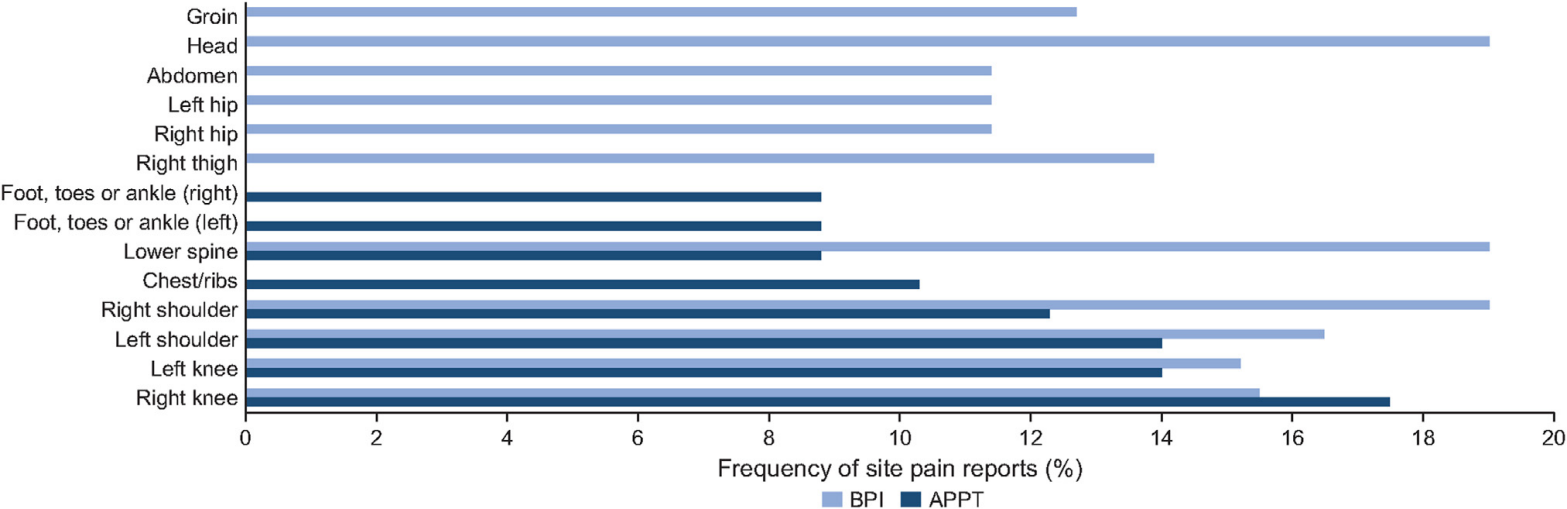
**Most common pain locations reported in the Adolescent Pediatric Pain Tool (APPT) and adult Brief Pain Inventory (BPI) questionnaires.** Most frequent pain site locations reported in the APPT (dark blue bars) and BPI (light blue bars) questionnaires by child/adolescent and adult populations. APPT, Adolescent Pediatric Pain Tool; BPI, Brief Pain Inventory.

Fifty-seven (98.3%) adolescents and all adult participants completed the pain questionnaires. Among the adolescents, 53.4% reported at least 1 pain site, and 10.3% reported 3 or more pain sites. For the adult population, 58 (73.4%) reported experiencing pain not described as everyday minor aches and pain. The most common locations of pain reported by the adolescents and adults are detailed in Figure 4. Pain in the right and left knees and left shoulder were the main complaints of the adolescents, whereas adults reported pain in the head, lower spine, and right shoulder (Figure 4). Similar results were observed in all countries for both child/adolescent and adult populations, except for the APPT temporal score, which was higher in Argentina, and scores for the BPI, which were lower in Colombia (Supplemental Figure 4).

### Burden of medical complications in pediatric population with achondroplasia

**Figure 5:**
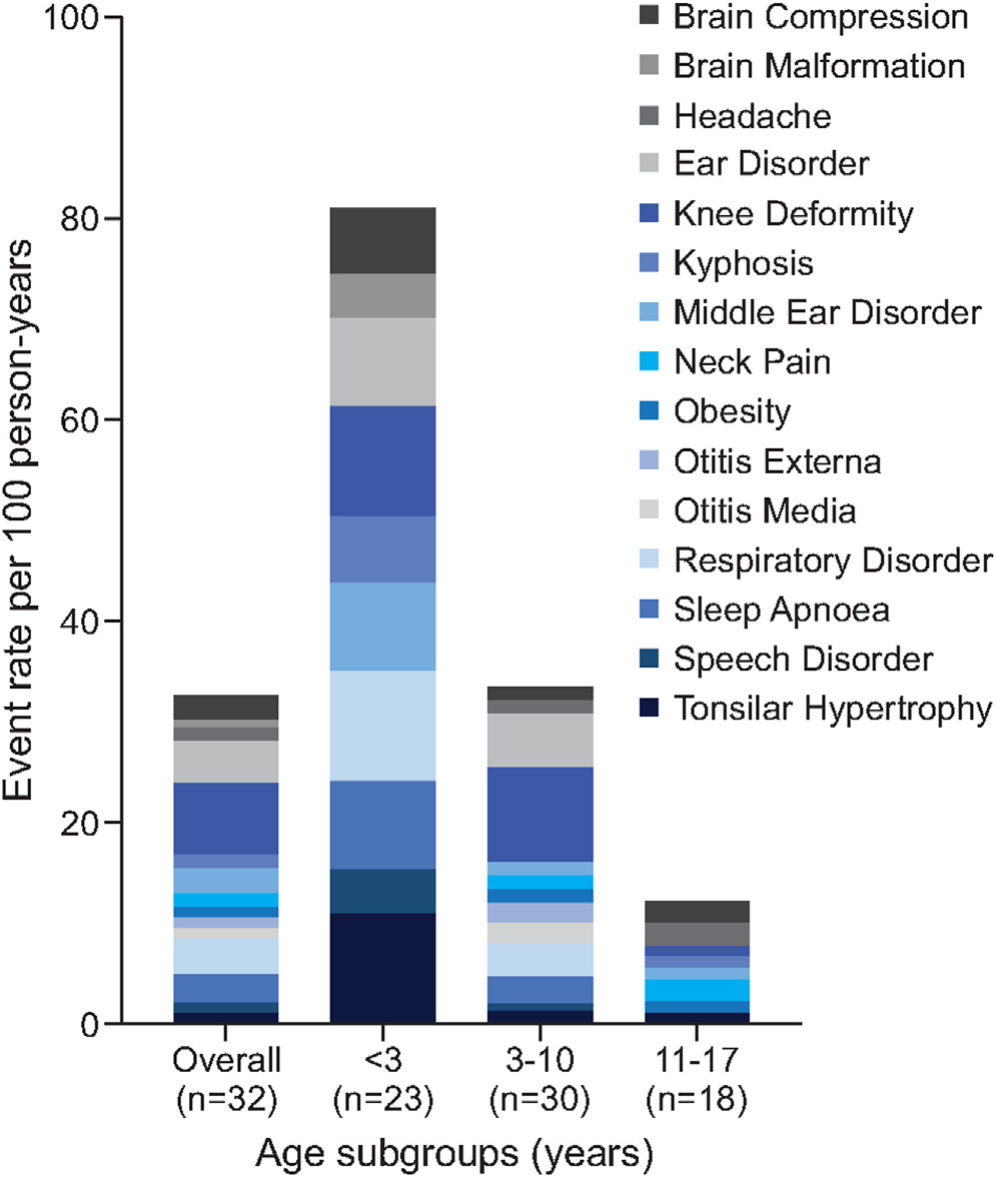
**Achondroplasia-related medical events by age group in the PHAS population.** Data are presented as event rates per 100 person-years, calculated as the total number of events divided by the total number of patient historical years multiplied by 100. Patients were included in the age groups according to the age at which the medical event was reported. Because each patient could have presented several events across their pediatric lifespan, they may be included in multiple age groups. Data related to ear disorders were collected from medical records during the COVID-19 pandemic, thus hampering onsite data gathering and harmonization by study monitors. To preserve information as they are classified in each site, we presented information separately and classified them in accordance with the MedRA categories (version 22.0).

Data from Argentinean achondroplasia pediatric population (*n* = 32 participants, the PHAS population) was analyzed separately. Almost all participants (*n* = 31, 96.9%) in this population had a history of achondroplasia-related medical events, with 130 events reported in 30 participants. The most common reported events by organ system classes reported (≥20%) were musculoskeletal and connective tissue disorders (accounting for 78.1% of the events), ear and labyrinth disorders (59.4%), infections (53.1%), thoracic disorders (50.0%), and nervous system disorders (46.9%). The events reported by their rate are shown in Figure 5. Except for knee deformity (the most commonly reported medical event, reported by 20 participants with an event rate of 7.02 per 100 person-years), the highest event rates were in the youngest age range (<3 years), with decreasing incidences observed in the older age groups (3-10 and 11-17 years) (Figure 5).

The majority of the pediatric participants, 28 (87.5%), reported a history of use of at least 1 medication, defined as any medications taken in the participants’ entire medical history. In line with the most reported medical events being related to musculoskeletal and connective tissue disorders (Figure 5), which were also reported as the main cause of pain (Figure 4), analgesics were the most prescribed class of medication—19 individuals (59.4%, event rate of 12.64) reported the use of this type of medication, followed by antibacterials for systemic use (*n* = 17, 53.1%, event rate 9.83), anti-inflammatories and antirheumatics (*n* = 17, 53.1%, event rate 9.48), corticosteroids for systemic use (*n* = 5, 15.6%, event rate 2.46), and drugs for obstructive airway diseases (*n* = 4, 12.5%, event rate 2.81). This pattern was consistent among the 3 age groups of the PHAS population (<3, 3-10 and 11-17 years).

This clinical burden caused by achondroplasia also led to high rates of medical resource use, which included medical examinations, health care professional (HCP) and in/outpatient hospital visits, and surgical procedures. Ten participants (31.3% of the PHAS population) reported 35 medical examinations in their medical history (Table 5). The age range with the most medical examinations was 3-10 years old (7 participants, 23.3%, event rate of 15.40) and an audiogram was the most common medical examination (Table 5), which correlates with the high rates of ear disorders reported among this population (Figure 5).

**Table 5:** Medical resource use of achondroplasia pediatric patients

Types of Medical Resource Use	Number of Events (Event Rate)^a^ by Age Group^b^			
	< 3 Years (*n* = 23)	3-10 Years (*n* = 30)	11-17 Years (*n* = 18)	Overall (*n* = 32)
Patient historical years^c^	45.69	149.38	89.85	284.91
Medical examination				
Total number of events (event rate per 100 person-years)	2 (4.38)	23 (23.3)	10 (11.13)	35 (12.28)
Audiogram	0	2 (1.34)	1 (1.11)	3 (1.05)
Biopsy	0	0	1 (1.11)	1 (0.35)
Brain stem auditory evoked response	0	0	1 (1.11)	1 (0.35)
Computerized tomogram head	0	2 (1.34)	0	2 (0.70)
Computerized tomogram spine	0	3 (2.01)	0	3 (1.05)
Health care professional visit				
Total number of visit events (event rate per 100 person-years)	9 (19.70)	9 (6.03)	5 (5.56)	23 (8.07)
ENT physician and/or surgeon	0	1 (0.67)	0	1 (0.35)
Occupational Therapist	0	1 (0.67)	0	1 (0.35)
Orthotist	0	1 (0.67)	0	1 (0.35)
Pediatrician	0	1 (0.67)	1 (1.11)	2 (0.70)
Physiotherapist	7 (15.32)	1 (0.67)	4 (4.45)	12 (4.21)
Inpatient hospital visit				
Total number of visit events (event rate per 100 person-years)	10 (21.89)	24 (16.07)	18 (20.03)	53 (18.60)
Foramen magnum syndrome/stenosis	1 (2.19)	2 (1.34)	0	3 (1.05)
Genu varum/valgum	0	2 (1.34)	5 (5.56)	7 (2.46)
Limb lengthening	0	0	6 (6.68)	6 (2.11)
Other	5 (10.94)	2 (1.34)	1 (1.11)	8 (2.81)
Other ENT issues	4 (8.76)	17 (11.38)	6 (6.68)	28 (9.83)
Pain	0	1 (0.67)	0	1 (0.35)
Outpatient hospital visit^d^				
Total number of visit events (event rate per 100 person-years	55 (120.38)	205 (137.24)	189 (210.36)	449 (157.59)
ENT physician and/or surgeon	6 (13.13)	30 (20.08)	22 (24.49)	58 (20.36)
Neurologist	1 (2.19)	9 (6.03)	14 (15.58)	24 (8.42)
Neurosurgeon	4 (8.76)	24 (16.07)	18 (20.03)	46 (16.15)
Orthopedist	8 (17.51)	44 (29.46)	37 (41.18)	89 (31.24)
Pediatrician	31 (67.85)	67 (44.85)	52 (57.88)	150 (52.65)

*ENT*, ear, nose, and throat.

a Event rates per 100 person-years were calculated as the total number of events divided by the total number of patient historical years multiplied by 100.

b Age group is based on age at time of event. Patients may be counted in multiple columns.

c Patient historical years are defined as the time between earliest collected history and enrollment date.

d Only the top 5 specialists (by total number of event rate) are listed.

All 32 patients in the PHAS population reported at least 1 HCP visit, with a total of 2177 events over 285 person-years of medical history. The list of the type of HCP visited is detailed in Table 5. Disease follow-up was the main reason for HCP visits (reported by 12 participants, overall event rate of 5.62), and a physiotherapist was the most visited HCP in all age groups (<3, 3-10 and 11-17 years), being more common in the < 3- and 11-17-year age groups (Table 5).

**Figure 6:**
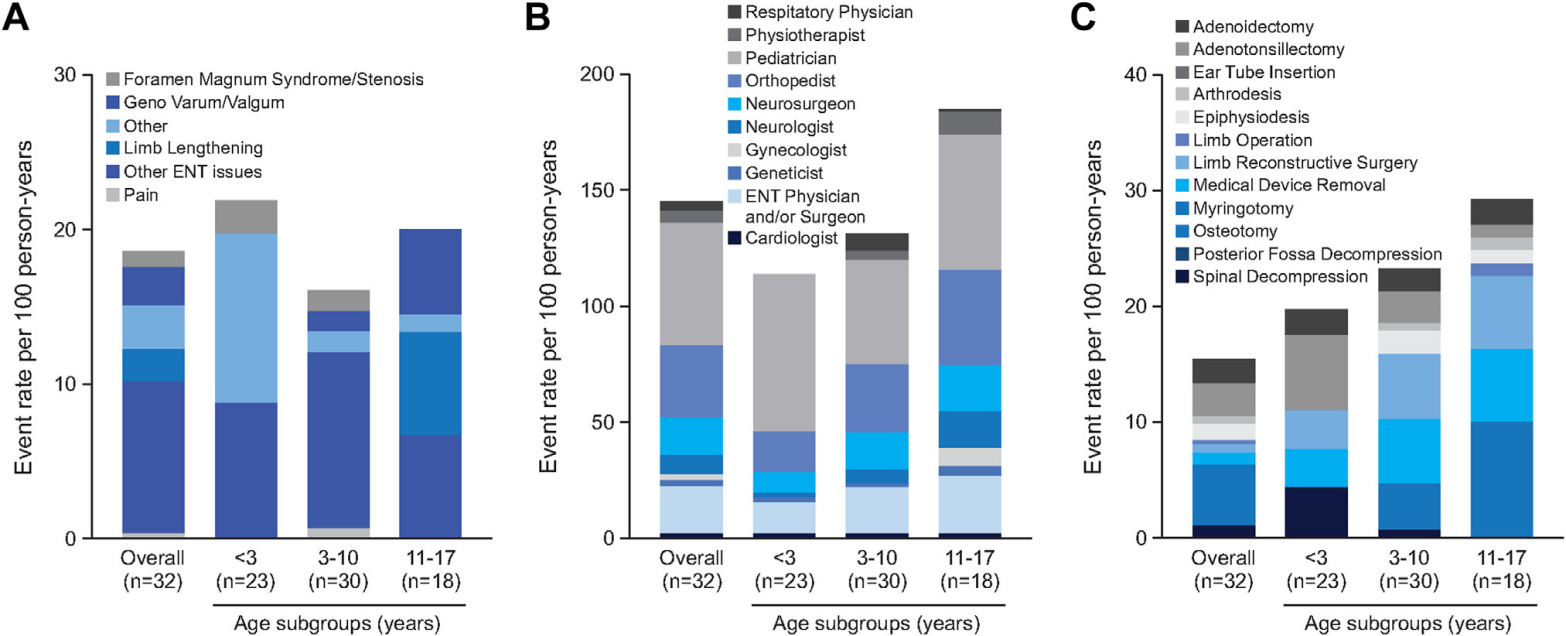
**History of medical resource use by age group for the PHAS population.** Data of inpatient (A) or outpatient (B) hospital visits and surgical procedures (C) are presented as event rates per 100 person-years, calculated as the total number of events divided by the total number of patient historical years multiplied by 100. For the hospital visits, a patient may have more than 1 visit coded to the same category but is counted only once per category. For the surgery history, patients were included in the age group according to the age at the time of procedure and the participants may be counted in multiple groups. The medical history is reported using he MedDRA preferred terms (version 22.0). ENT, ear, nose, and throat.

Moreover, 71.9% of participants reported an inpatient visit, with 53 events occurring in 23 patients (event rate 18.60). “Other ENT issues” are listed as the most common reason for inpatient hospital visits, with an event rate of 9.83 in 13 participants, occurring mainly in the 3-10 age range (Table 5 and Figure 6A). “Other” (including adenotonsillectomy, sleep studies, gastrointestinal disorders, and ventriculoperitoneal shunt surgery) was the second most reported event, with an event rate of 2.81 (*n* = 6 participants), followed by genu varum/valgum, with an event rate of 2.46 (*n* = 6) (Table 5 and Figure 6A). Twenty-eight participants (87.5%) reported outpatient hospital visits with 448 events, with higher total number of events being reported in the age ranges of 3 to 10 (205) and 11 to 17 (189) years (Table 5 and Figure 6B). Pediatricians (150 events in 27 participants) and orthopedists (89 events in 22 participants) were the specialists most visited during outpatient visits (Table 5 and Figure 6B).

Twenty-four (75%) participants reported a surgery with 54 events across the 285 person-years of medical history, the highest incidence being in the 3 to 10 age group (Figure 6C). The event rate of surgical procedures increases with the age groups and the highest was found in the 11-to-17-year age group (*n* = 11 participants, event rate 22.26) (Figure 6C). As expected for achondroplasia patients, younger children (<3 years) most commonly underwent adenotonsillectomy (event rate 6.57, with 3 participants reporting it over a relatively short time period) and spinal decompression procedures (Figure 6C). From age 3 to 10 years, the most common surgeries were adenotonsillectomy and myringotomy (tied for the highest event rate of 2.68, reported by 4 participants each), and surgeries related to limbs and osteotomy were also reported. In the 11-to-17-year age group, the highest event rate was for osteotomy at 5.56, reported by 4 participants. The 3 patients who underwent limb lengthening surgery reported at least 1 surgical event, with a total of 12 events reported. All of them underwent osteotomy surgery.

## Discussion

LISA was the largest retrospective, observational, cross-sectional study assessing HRQoL ever undertaken that set out to characterize and quantify the lifetime impact of achondroplasia on patients in LATAM. The study was conducted at 4 sites in 3 countries, 2 sites in Brazil, 1 in Colombia, and 1 in Argentina. The COVID-19 pandemic made the recruitment of participants (particularly in Brazil) and the ability to source the complete medical record of the patients difficult. Despite this, 172 of the planned 175 participants were enrolled, and all completed the study. The sample presented a broad age range, with median age of 16.0 years, and were homogenous across the countries and the data analysis sets, with exception of the PHAS, which only comprised pediatric patients. Individuals were diagnosed early, with a median age of diagnosis of 0.7 months.

Achondroplasia-related symptoms, complications, and comorbidities greatly affect patients’ physical functioning and psychosocial life, leading to a poorer QoL in some areas when compared with average stature people. Regardless of the age group and country, children and adolescents from the LISA cohort presented suboptimal HRQoL parameters, as measured by lower scores in several domains of the QoLISSY and PedsQL instruments. The overall QoLISSY scores were generally in the 60 to 70 range, indicating that HRQoL was below average compared with the reference population of average stature individuals (total QoLISSY score around 82). The lowest individual scores in the QoLISSY were in respect of the physical and social domains, which reflects the multiple challenges that patients have to face and the associated reduced accessibility and independence, reduced self-esteem, and socialization issues.^13^, ^14^, ^15^ Parent-reported scores were generally lower than the patients’ self-reported scores in the QoLISSY questionnaire, indicating that parents perceive a more negative impact of the disease in their children QoL than the patients themselves. Other studies also demonstrated this trend.^17^^,^^23^

The Lifetime Impact of Achondroplasia in Europe (LIAISE), a similar study recently undertaken with European patients with achondroplasia in 6 countries (Denmark, Austria, Germany, Sweden, Spain, and Italy) also showed reduced scores across most domains of the QoLISSY and PedsQL questionnaires.^17^ However, emotional functioning was rated high based on the QoLISSY instrument in both LISA and LIASE studies, indicating good mental/emotional-related QoL. Good emotional health was also observed for patients with other chronic medical conditions because of their improved ability to accept themselves and to deal with daily stressors in comparison with healthy children^23^^,^^24^ in addition to the attention and assistance received from achondroplasia supportive organizations.^15^^,^^17^

The other questionnaires also revealed impairments in the childhood and adolescent age groups through measurements of functionality (WeeFIM) and pain (APPT). WeeFIM scores were similar across all domains and increased with age, indicating greater independence in older patients. Previous studies evaluating independence in children with achondroplasia suggest that the patients’ shortened limbs decrease the functionality of their motor skills and delay their acquisition of developmental milestones, leading to lower scores, especially up to 7 years old, an age range when the children usually start to require less assistance from their parents/caregivers in respect of self-care, such as showering, brushing teeth, and getting dressed.^14^^,^^15^^,^^22^ The reduced motor functionality is also correlated to pain, and, indeed, our results from the APPT questionnaire revealed that more than 50% of adolescent participants experienced at least 1 pain site, mainly in the knees and shoulders. The LIAISE study reported 3 times more participants with 3 or more pain sites and higher total pain scores.^17^

Pain has also been correlated with impaired physical function in adults.^14^ Ten percent of adults included in LISA reported 3 or more pain sites, with the lower spine, shoulder, knees, and thigh being the most cited sites. The majority of the adult population classified the pain as moderate or severe. These findings are consistent with the reports of adult patients experiencing difficulties in standing for long periods, reaching for things, and losing energy in the NHP inventory.^8^^,^^9^ Other studies correlated increased musculoskeletal pain in late adulthood with the poor QoL reported by older age groups when compared with average-statured individuals and also with children/adolescent/young adults with achondroplasia.^14^ In LISA, despite the pain, adults’ overall health status was comparable to the reference population, as demonstrated by the comparable EQ-5D-5L index scores. The adult self-rated mean EQ-5D-5L VAS score was lower than the unaffected population, and patients in Brazil reported lower scores than patients from the other LATAM countries participating in the LISA study. This may be related to the anxiety and depression subscale, where higher rates were reported in Brazil. Indeed, patients diagnosed with some psychiatric illness, which represented the majority of a US cohort of adults with achondroplasia, also showed worse physical and mental health status.^25^ The NHP reported scores were considered high, indicating poor subjective health status in the adult population, being worse for those who had undergone limb lengthening surgery.

The risk-benefit of performing limb lengthening procedures are controversial among patients with achondroplasia, caregivers and physicians in terms of height gain and associated risks and complications.^15^^,^^26^^,^^27^ Previous studies that evaluated the effects of lower limb lengthening surgery on physical functioning and QoL assessments in patients with achondroplasia were inconclusive.^14^ There were few reports of limb lengthening in the LISA cohort, consistent with other recent reports in the literature for LATAM.^4^ The majority of limb lengthened participants were from Colombia, which could be because the Colombian site was coordinated by an orthopedic surgeon. Whereas participants who underwent limb lengthening were more likely to report pain in the knee, lower spine, foot/toes/ankle, and stomach/abdomen, it seems that their functionality and independence tended to improve. However, it is unclear whether this was related to the effects of the limb lengthening surgery itself or the fact that patients undergoing limb lengthening may have had better overall health supervision, leading to better medical care focused on achondroplasia.

Historical medical data from the Argentinean pediatric patients with achondroplasia (PHAS population) were analyzed separately because this was the only site that had access to comprehensive medical records over the reported period. This generated more robust data on medical resource use in the pediatric population. However, the challenges with obtaining medical records for the duration of the retrospective medical history at the other sites means that we cannot make conclusions about the medical burden of achondroplasia in those countries. The main achondroplasia-related medical events reported were in the class of musculoskeletal and connective tissues (78.1% of the events) and ENT disorders (59.4%), knee deformity being the most commonly reported (7.02 event rate per 100 person-years) in the lower age subgroups (<3 and 3-10 years old). These types of event are expected in the achondroplasia population^7^, ^8^, ^9^ and were also observed in the LIAISE study.^17^ Both LISA and LIAISE studies found high rates of a broad variety of disease-related complications, particularly at young ages, corroborating with the morbidity rates and prevalence of the multisystemic effects of achondroplasia.^9^, ^10^, ^11^^,^^28^ The medical events reported also correlate to the most used medication classes, which were analgesics, anti-inflammatory and antirheumatics.

The high rates of ear disorders in the pediatric population made audiograms the most commonly reported medical examination in <10 years old population. High frequency of HCP visits was found, and several HCP specialties were visited, especially physiotherapists, pediatricians, ENT physicians, and/or surgeons, highlighting the multidisciplinary disease follow-up (also described as the main reason for HCP visits). Hospital visits were generally because of ENT issues, genu varum/valgum, and limb lengthening procedures. With respect to surgeries, 75% of the PHAS population reported at least 1 surgery and the most common types of surgery were adenotonsillectomy, myringotomy, adenoidectomy, and osteotomy, which are consistent with the pathology of achondroplasia and in accordance to the practical guidelines of management of ENT complications requiring surgery.^29^^,^^30^ These data suggest the burden of the disease in younger patients intensifies the need for health system use.

Despite the limitations of the LISA study, namely, the differences between the investigator specialties and treatment models of the sites participating in the study, and the diversity of patients enrolled, the results of the assessments undertaken using the HRQoL, WeeFIM, and pain questionnaires were similar across the sites, indicating that patients living with achondroplasia experience a significant burden of illness across multiple domains and a reduced QoL compared with healthy individuals. The LISA data showed that achondroplasia is associated with multisystem complications, pain, and discomfort, which affect QoL, functionality, and the independence of individuals living with the disease.

## Conclusions

The LISA study addressed gaps in the knowledge about the clinical and socioeconomic burden of the illness, HRQoL, the psychosocial impact, and health care resource use, and provided the largest data set to date of individuals with achondroplasia in Latin America. Although this study was not a longitudinal lifetime impact study, the 3-year assessment of retrospective and cross-sectional data and information collected from the patient-reported outcomes in respect of QoL and psychosocial capability demonstrated that patients with achondroplasia experience a significant burden of the disease. These data can help to improve the overall understanding of the impact of achondroplasia on the individuals living with disease, supporting the development of regional strategies to increase access to medical care and adjust management practices as a way to improve patients HRQoL by decreasing the existing burden, especially with increased availability of disease specific treatment options.

## Data Availability

The data sets for the study presented in this publication may be available from the corresponding author upon request.

## ORCIDs

Juan Llerena Jr: http://orcid.org/0000-0002-4308-3841

Pablo Rosselli: http://orcid.org/0000-0003-0409-6583

Amanda Aragão: http://orcid.org/0009-0008-1740-9837

Cristina Valenzuela: http://orcid.org/0009-0001-0804-8258

Debora Bertola: http://orcid.org/0000-0002-4701-6777

Yaneth Mendez: http://orcid.org/0009-0001-5265-5768

Mariana del Pino: http://orcid.org/0000-0001-6095-7080

Nicolette Calvacanti: http://orcid.org/0009-0002-0892-4147

Paula Thomazinho: http://orcid.org/0000-0003-1214-563X

Jeanne M. Pimenta: http://orcid.org/0000-0003-3482-7770

Shelda Cohen: http://orcid.org/0009-0002-0597-3568

Tom Butt: http://orcid.org/0000-0002-0387-4550

José C. Thomaz Jr: http://orcid.org/0009-0002-4507-8162

Renée Shediac: http://orcid.org/0000-0001-6468-7866

Richard Rowell: http://orcid.org/0000-0002-6584-3389

Tatiana S.P.C. Magalhães: http://orcid.org/0000-0002-3904-2238

Chong Kim: http://orcid.org/0000-0002-1754-1300

Virginia Fano: http://orcid.org/0000-0003-3311-2220

## Conflict of Interest

Juan Llerena Jr has received consultancy and research grants, speaker fees, and travel support from BioMarin. Pablo Rosselli has received speaker fees and travel support from BioMarin. Amanda Aragão has received travel support from BioMarin. Debora Bertola has participated as a clinical trial investigator. Mariana del Pino has received speaker fees from BioMarin and participated as a clinical trial investigator. Nicolette Calvacanti has received research grants from BioMarin. Jeanne M. Pimenta, Shelda Cohen, Tom Butt, José C. Thomaz Jr, Renée Shediac, Richard Rowell, and Tatiana S.P.C. Magalhães, who are full-time employees of BioMarin, hold stocks in the company. Chong Kim has received travel support from BioMarin. Virginia Fano has received speaker fees from BioMarin and participated as a clinical trial investigator. All other authors declare no conflicts of interest.
