# Supplementary material for: Lifetime Impact Study for Achondroplasia (LISA): Findings from an observational and multinational study focused on health-related quality of life in individuals with achondroplasia in Latin America
Source: Genet Med Open. 2023 Nov 18;2:100843. doi: 10.1016/j.gimo.2023.100843 (PMC11613863; doi:10.1016/j.gimo.2023.100843)
Supplement: Supplementary Material [file mmc1.docx]

**Lifetime Impact Study for Achondroplasia (LISA): Findings from an observational and multinational study focused on health-related quality of life in individuals with achondroplasia in Latin America**

**Author names**

Juan Llerena Jr. ^1^, Pablo Rosselli ^2^, Amanda Aragão ^1^, Cristina Valenzuela ^2^, Debora Bertola ^3^, Yaneth Mendez ^2^, Mariana del Pino ^4^, Nicolette Calvacanti ^1^, Paula Thomazinho ^1^, Jeanne M. Pimenta ^5^, Shelda Cohen ^5^, Tom Butt ^6^, José C Thomaz Jr ^6^, Renée Shediac ^6^, Richard Rowell ^6^, Tatiana SPC Magalhães ^7^, Chong Kim ^3^, Virginia Fano ^4^

**Affiliations**

^1^ Instituto Fernandes Figueira, Fundação Oswaldo Cruz, Rio de Janeiro, Brazil

^2^ Fundación Cardioinfantil, Instituto de Cardiología, Bogotá, Colombia

^3^ Instituto da Criança, Hospital das Clínicas da Faculdade de Medicina da Universidade de São Paulo, São Paulo, Brasil

^4^ Hospital de Pediatría “Prof. Dr. Juan P. Garrahan”, Buenos Aires, Argentina

^5^ BioMarin (U.K.) Limited, London, UK

^6^ BioMarin Pharmaceutical Inc, Novato, CA, USA

^7^ BioMarin Farmacêutica do Brasil Ltda, São Paulo, Brazil

**SUPPLEMENTAL MATERIAL**

**Table S1. Child/Adolescent and adult questionnaires applied in the study with the outcome domains assessed.**

| **Questionnaire** | **Quality of Life** | **Physical Function** | **Psychosocial Function** | **Pain** | **Emotional/ Coping** | **Activity Impact** |
| --- | --- | --- | --- | --- | --- | --- |
| **Pediatric** | | | | | | |
| PedsQL*^a^* | × | × | × |  | × |  |
| QoLISSY *^a^* | × | × | × |  | × |  |
| WeeFIM |  | × |  |  |  | × |
| APPT |  |  |  | × |  |  |
| **Adult** | | | | | | |
| EQ-5D- 5L*^a^* | × | × | × | × |  | × |
| NHP | × | × | × | × | × |  |
| BPI-SF |  |  |  | × |  |  |
| WPAI |  |  |  |  |  | × |

*^a^* Values for reference population available (average stature individuals); **Abbreviations**: PedsQL, Pediatric Quality of Life Inventory Questionnaire ^1^; QoLISSY, Quality of Life in Short Stature Youth ^2^; WeeFIM, Pediatric Functional Independence Measure ^3,4^; APPT, Adolescent Pediatric Pain Tool ^5^; EQ-5D-5L, 5-level EQ-5D version of EuroQol ^6^; NHP, Nottingham Health Profile; BPI-SF, Brief Pain Inventory – Short Form; WPAI, Work Productivity and Activity Impairment.

**Supplemental Figure 1**

**
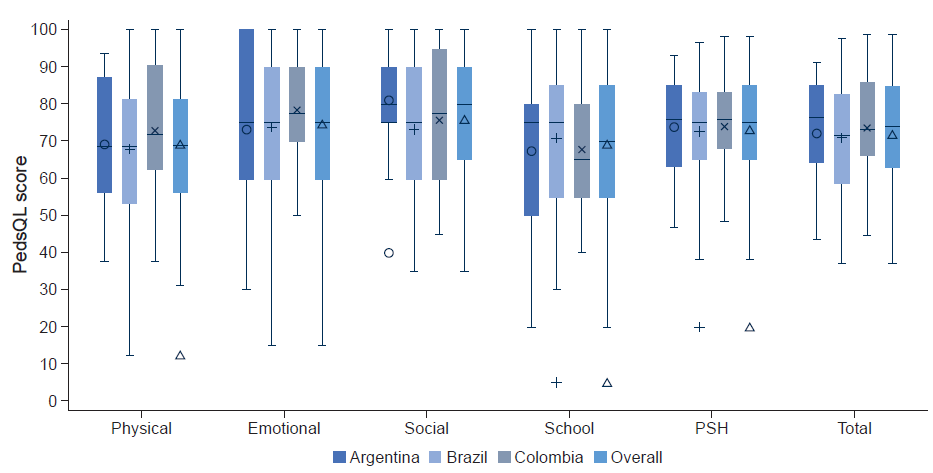
**

**Figure S1. Pediatric Quality of Life Inventory (PedsQL) scores by country.** Individual domains (physical, emotional, social, and school functioning) and total score of the child version of the PedsQL questionnaire divided by country. The Psychosocial Health (PSH) Summary Score is defined as the mean score for each item involved in the emotional, social, and school domains. The scores range from 0 (poor health-related quality of life) to 100 (better health-related quality of life). The figure is a Box-and-Whisker Plot. The score for each domain represents the mean score for each item involved in the domain. The whiskers are drawn from the quartiles (Q1 and Q3) to the extreme values (upper and lower limits). The symbol within the box stands for the mean, the middle line stands for the median. Outliers are defined as above 1.5 interquartile range.

**Supplemental Figure 2**


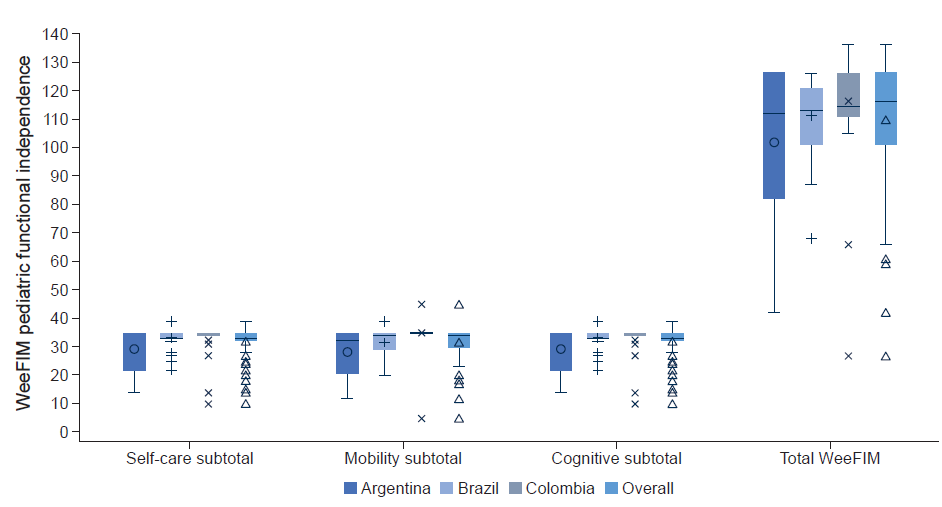


**Figure S2. Pediatric Functional Independence Measure (WeeFIM) by country for the child/adolescent achondroplasia patients.** Achondroplasia child/adolescent population (from 5 to 17 years) scores related to individual (self-care, mobility and cognitive) and total WeeFIM rating. Lower scores indicate “total assistance” and, higher scores, indicate “complete independence”. Self-care score ranges from 8 to 56; mobility and cognition scores range from 5 to 40; and the total WeeFIM score ranges from 18 to 126. The figure is a Box-and-Whisker Plot. The whiskers are drawn from the quartiles (Q1 and Q3) to the extreme values (upper and lower limits). The symbol within the box stands for the mean, the middle line stands for the median, the symbols outside of the box stands for outlier, if any. Outliers were defined as above 1.5 interquartile range.

**Supplemental Figure 3**


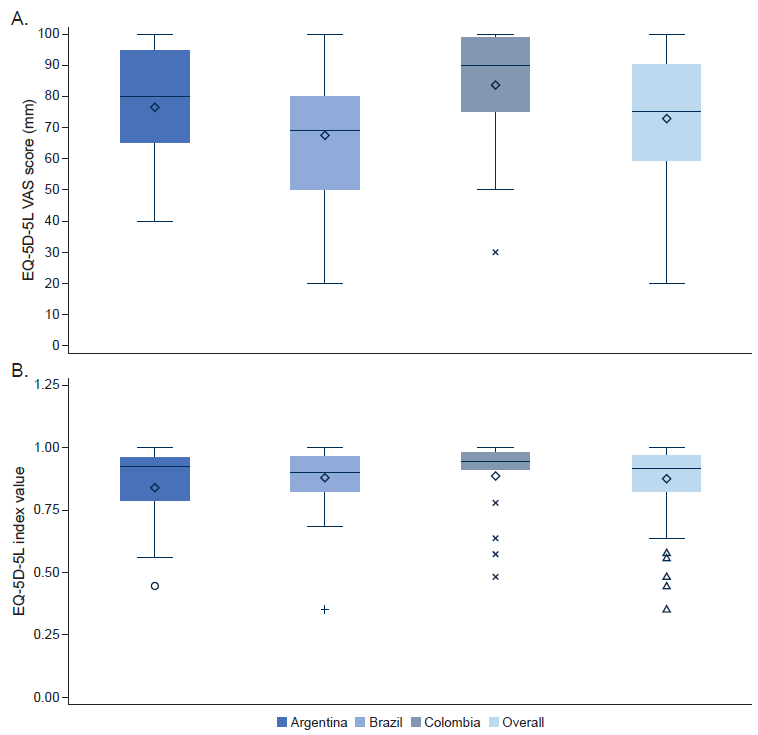


**Figure S3. EuroQoL (EQ-5D-5L) scores by country for the achondroplasia adult population.** The EQ-5D-5L was evaluated in adults by VAS scores (A, overall self-rated health status) and Index values (B, overall health status). The VAS score ranged from 0 (poor health status) to 100 (better health status) and individual domains score (Mobility, Self-care, Usual Activities, Pain/Discomfort, and Anxiety/Depression) quoted on 5 levels: no problems, slight problems, moderate problems, severe problems, and extreme problems. The index score ranged from around -0.6 (poor health) to 1.0 (better health). The figure is a Box-and-Whisker Plot. The whiskers are drawn from the quartiles (Q1 and Q3) to the extreme values (upper and lower limits). The symbol within the box stands for the mean, the middle line stands for the median, the symbols outside of the box stands for outlier, if any. Outliers are defined as above 1.5 interquartile range.

**Supplemental Figure 4**


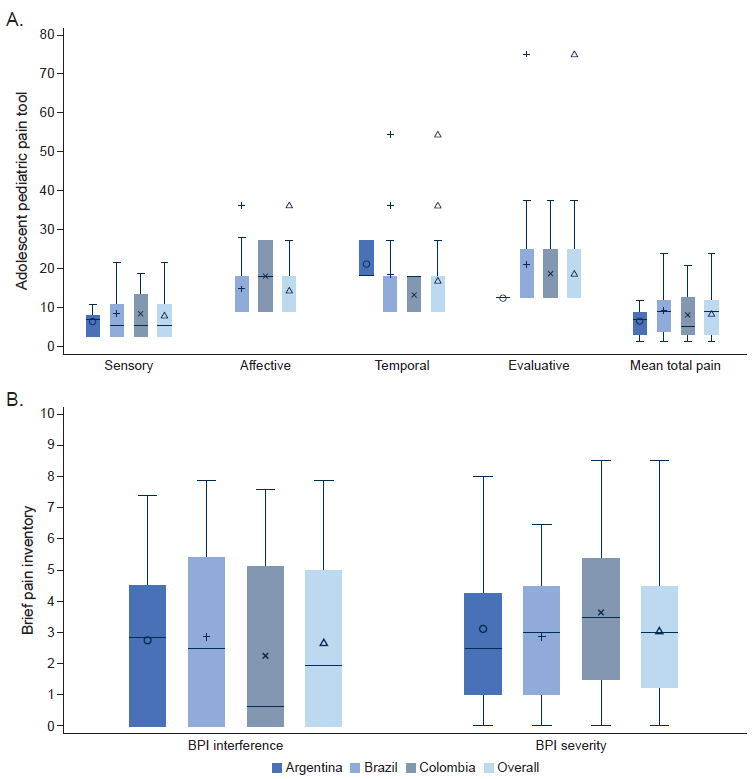


**Figure S4. Adolescent Pediatric Pain Tool (APPT) and adult Brief Pain Inventory (BPI) questionnaire results by country.** APPT (A) are presented as percentage for the individual domains (sensory, affective, evaluative and temporal) and total pain score. In the BPI (B) questionnaire, pain severity score ranges from 0 (no pain) to 10 (pain as bad as you can imagine) and pain interference score ranges from 0 (does not interfere) to 10 (completely interferes). Figures are Box-and-Whisker Plot. The whiskers are drawn from the quartiles (Q1 and Q3) to the extreme values (upper and lower limits). The symbol within the box stands for the mean, the middle line stands for the median, the symbols outside of the box stands for outlier, if any. Outliers were defined as above 1.5 interquartile range.

**References:**

1. PedsQL TM (Pediatric Quality of Life Inventory TM). Accessed November 24, 2022. https://www.pedsql.org/

2. Bullinger M, Quitmann J, Power M, et al. Assessing the quality of life of health-referred children and adolescents with short stature: development and psychometric testing of the QoLISSY instrument. *Health Qual Life Outcomes*. 2013;11:76. doi:10.1186/1477-7525-11-76

3. Serghiou MH, Rose MW, Pidcock FS, et al. The WeeFIM [R] instrument--a paediatric measure of functional independence to predict longitudinal recovery of paediatric burn patients. *Dev Neurorehabil*. 2008;11(1):39-50. doi:10.1080/17518420701520644

4. Ireland PJ, McGill J, Zankl A, et al. Functional performance in young Australian children with achondroplasia. *Dev Med Child Neurol*. 2011;53(10):944-950. doi:10.1111/j.1469-8749.2011.04050.x

5. PhenX Toolkit: Protocols. Accessed November 24, 2022. https://www.phenxtoolkit.org/protocols/view/190902

6. EQ-5D-5L – EQ-5D. Accessed November 24, 2022. https://euroqol.org/eq-5d-instruments/eq-5d-5l-about/
